# Multisystem Inflammatory Syndrome in Children after Coronavirus Disease 2019 and Its Cytokine Storms

**DOI:** 10.31083/j.rcm2412371

**Published:** 2023-12-27

**Authors:** Keiichi Hirono

**Affiliations:** ^1^Department of Pediatrics, Faculty of Medicine, University of Toyama, 930-0194 Toyama, Japan

Cardiac injury in coronavirus disease 2019 (COVID-19) is probably the second leading cause of infection-related mortality. Various cardiac abnormalities have been reported in pediatric COVID-19 cases, including ventricular dysfunction, acute myocardial injury, arrhythmias, conduction abnormalities, coronary artery dilation and aneurysms, and occasionally pericarditis and valvulitis. Given that the mechanisms underlying myocardial injury associated with COVID-19 include cardiomyocyte cell injury caused by a severe acute inflammatory response during cytokine storms, cardiomyocyte cell injury caused by viral invasion, ischemic injury associated with severe hypoxia due to acute lung injury, and procoagulant states, an algorithm evaluating the risk of thrombosis and inflammatory status in pediatric patients with COVID-19 is needed. Balas *et al*. [1] evaluated the importance of cardiac monitoring in patients with COVID-19 and highlighted its characteristics in pediatric patients.

Since 2020, cases of multisystem inflammatory syndrome in children (MIS-C) associated with COVID-19 have been reported in the relevant literature [2]. MIS-C is a disorder that causes inflammation in various parts of the body, including the heart, lungs, kidneys, brain, skin, eyes, and gastrointestinal system. The features of this condition are similar to those of Kawasaki disease in approximately half of the cases. Studies from abroad have reported that the incidence rate of MIS-C is 0.03% after severe acute respiratory syndrome coronavirus 2 (SARS-CoV-2) infection. Among patients with multisystem inflammatory syndrome in children/pediatric inflammatory multisystem syndrome (MIS-C/PIMS), 73.3% and 3.8% required treatment in pediatric intensive care units and extracorporeal membrane oxygenation, respectively [3]. Despite the severity of MIS-C/PIMS, its mortality rate has been reported to be 0.9%. Notably, MIS-C primarily affects school-aged children with a median age of approximately 8 years, whereas Kawasaki disease typically affects children with an age of approximately 1 year. It affects 55% males and is more common among African, Hispanic, and White populations than among Asian populations [4]. Common clinical manifestations of MIS-C include fever (99% cases), shock (32%–76% cases), gastrointestinal symptoms (vomiting, diarrhea, and abdominal pain [87% cases]), neurological symptoms (headache, lethargy, and confusion [36% cases]), and mucocutaneous symptoms (primarily polymorphic rash [59% cases], limb edema [9%–16% cases], conjunctival congestion [57% cases], cervical lymph node swelling [25% cases], and mild respiratory symptoms [40% cases]). In particular, gastrointestinal symptoms such as abdominal pain (70% cases), vomiting (60% cases), and diarrhea (57% cases) are frequently observed. Meanwhile, respiratory symptoms are less common and usually mild. Compared with Kawasaki disease, MIS-C has fewer skin manifestations, such as limb edema, strawberry tongue, and cervical lymph node swelling. Approximately 36% of MIS-C cases meet the diagnostic criteria for Kawasaki disease, with 6% and 30% being typical and incomplete cases, respectively [5]. Key distinguishing features between MIS-C and Kawasaki disease include older age at onset of MIS-C, severe circulatory impairment, and severe abdominal pain, often associated with fulfillment of fewer than four of the diagnostic criteria of Kawasaki disease. Laboratory findings of MIS-C resemble those of Kawasaki disease, with 89%–95% and 31%–80% cases exhibiting lymphopenia and thrombocytopenia, respectively.

There was a temporal delay of several weeks between the peak of the COVID-19 pandemic and the increase in MIS-C cases, suggesting that MIS-C resulted from a delayed abnormal immune response after SARS-CoV-2 infection [6]. Many children with MIS-C develop symptoms 2–6 weeks after SARS-CoV-2 infection and often exhibit polymerase chain reaction negativity at the time of presentation. Various inflammatory cytokines, such as interferon gamma (IFN-γ), interleukin-10 (IL-10), IL-6, IL-8, chemokine interferon-γ inducible protein 10 kDa (CXCL10), macrophage inflammatory protein-1α (MIP-1α), MIP-1β, tumor necrosis factor alpha (TNF-α), and IL-17, are increased in patients with MIS-C compared with healthy individuals and pediatric patients with COVID-19 (Table 1) [7]. In cases of MIS-C and myocarditis, significant elevations are observed in IFN-γ, IL-18, and interferon γ-induced protein 10 kDa (IP-10), which are not as prominent in other patients with MIS-C or Kawasaki disease. These cytokines and chemokines are largely associated with the nuclear factor kappa B and TNF-α signaling pathways. Increased levels of chemokines such as C-C motif chemokine ligand 2 (CCL2), CCL3, CCL20, CX3CL1, and CXCL10 may promote cell migration into inflamed tissues. Moreover, it has been reported that these levels can cause inflammatory monocyte (CD16+) activation [7]. These findings suggest that IFN-γ and inflammatory monocytes are the primary drivers of inflammation in MIS-C.

**Table 1.** **Cytokines reported to have variations in MIS-C**.

Classification	Cytokines	Main producing cells	Main functions
IFN-γ	IFN-γ	NK cells, T cells	Macrophage activation, Th1 induction, antigen-presenting cell activation
TNF-α	TNF-α	Macrophages, NK cells, T cells	Activation of endothelial cells, neutrophils
Interleukin	IL-6	B cells, endothelial cells, macrophages, T cells	Cell cycle promotion, lymphocyte and stromal cell activation, induction of acute-phase proteins
	IL-8	MΦ, epithelial cells, airway smooth muscle cells, endothelial cells	Chemotaxis of neutrophils
	IL-10	Th2 cells	Inhibition of cytokine signaling and transcription
	IL-17	CD4 T cells	Activation of inflammatory cells such as endothelial cells and macrophages
	IL-18	Dendritic cells, endothelial cells, monocytes, fibroblasts, macrophages	Promotion of endothelial cell and macrophage proliferation, inflammatory response, IFN-γ production
	IL-2RA	T cells, B cells, macrophages, dendritic cells	T-cell proliferation and activation, B-cell proliferation and enhanced antibody production, activation of monocytes/macrophages, proliferation/activation of NK cells, induction of LAK cells
Chemokine	MIP-1α	Macrophages	Selective chemotaxis of CD8+ lymphocytes
	MIP-1β	Macrophages	Selective chemotaxis of CD4+ lymphocytes
	CXCL9	Monocytes, macrophages, endothelial cells	Chemotaxis of Th1 lymphocytes, inhibition of tumor cell proliferation, angiogenesis, inhibition of colony formation of hematopoietic precursor cells
	CXCL10	Monocytes, endothelial cells, fibroblasts	Chemotactic induction for monocytes, macrophages, T cells, NK cells, dendritic cells, adhesion of T cells to endothelial cells, anticancer activity, colony formation, angiogenesis in the spinal cord
	IP-10	Monocytes, endothelial cells, fibroblasts	Chemotaxis of dendritic cells, monocytes, macrophages, NK cells, T cells

MIS-C, multisystem inflammatory syndrome in children; IFN-γ, interferon gamma; IL, interleukin; CXCL10, chemokine interferon-γ inducible protein 10 kDa; MIP-1α, macrophage inflammatory protein-1α; IP-10, interferon γ-induced protein 10 kDa; TNF-α, tumor necrosis factor alpha; NK, natural killer; LAK, lymphkine activated killer; MIP-1β, macropahge inflammatory protein -1 beta; IL-2RA, interleukin 2 receptor subunit alpha; CXCL9, CXC motif chemokine ligand 9.

Compared with Kawasaki disease, in MIS-C, naive CD4+ cells decrease, whereas central memory and effector memory CD4+ T cells increase [8]. In a previous study, T-cell repertoire analysis revealed that the proliferation of Vβ21.3+ CD4 and CD8 T cells is specific to patients with MIS-C compared to patients with Kawasaki disease, patients with toxic shock syndrome, and adult patients with COVID-19 [9]. The expansion of Vβ21.3+ T cells is correlated with elevated levels of the serum cytokines IL-18 and IL-1Ra and is associated with a highly activated phenotype characterized by high expression of HLA-DR and CD38, along with high expression of CX3CR1—a marker for monocytes and cytotoxic lymphocytes. CX3CR1 is a membrane-bound chemokine induced in vascular endothelial cells during inflammation and plays an important role in vascular inflammation.

The late-onset inflammation observed in cases of MIS-C cannot be solely explained by the release of inflammatory mediators after T-cell activation and cytokine release. Evidence suggests the presence of B-cell autoimmune responses in cases of MIS-C. Moreover, autoantibodies have been detected in the mucous membranes, heart tissues, endothelial cells, and cytokine molecules of patients with MIS-C [10]. Further, these patients exhibit other immunological events that are consistent with virus-induced autoimmunity, such as the expansion of plasma cells and persistence of functional SARS-CoV-2-specific monocyte-activating antibodies [9]. In MIS-C, the presence of autoantibodies suggests a direct cross-reactivity between SARS-CoV-2 antigens and self-antigens, potentially contributing to the onset of the disease. In addition to B-cell autoimmune responses, antibody-dependent enhancement (ADE) may lead to MIS-C. Notably, ADE may exacerbate viral infections and immunopathology by causing excessive Fc-mediated effector functions and the formation of immune complexes, as seen in respiratory syncytial viral infections and measles. Children initially exposed to SARS-CoV-2 produce both neutralizing and non-neutralizing antibodies, with predominantly neutralizing antibodies leading to asymptomatic cases. However, a subset of children may form complexes of non-neutralizing antibodies and viral antigens, potentially leading to ADE and progression to severe MIS-C [11].

Thus, in MIS-C, IFN-γ appears to be a major factor in inflammation, and there is a possibility of ADE and increased adaptive immune responses. MIS-C affects both antigen and host responses, which can lead to prolonged antigen stimulation and chronic adaptive immune responses.

## Abbreviations

MIS-C, multisystem inflammatory syndrome in children; SARS-CoV-2, severe acute respiratory syndrome coronavirus 2; PIMS, pediatric inflammatory multisystem syndrome; IFN-γ, interferon gamma; IL, interleukin; CXCL10, chemokine interferon-γ inducible protein 10 kDa; MIP-1α, macrophage inflammatory protein-1α; TNF-α, tumor necrosis factor alpha; IP-10, interferon γ-induced protein 10 kDa; ADE, antibody-dependent enhancement; MIP-1β, macropahge inflammatory protein-1 beta.

## References

[1] Balas RB, Meliț LE, Mărginean CO (2023). COVID-19 and Cardiac Implications—Still a Mystery in Clinical Practice. *Reviews in Cardiovascular Medicine*.

[2] Forrest K, Weismer G, Milenkovic P, Dougall RN (1988). Statistical analysis of word-initial voiceless obstruents: preliminary data. *The Journal of the Acoustical Society of America*.

[3] Payne AB, Gilani Z, Godfred-Cato S, Belay ED, Feldstein LR, Patel MM (2021). Incidence of Multisystem Inflammatory Syndrome in Children Among US Persons Infected With SARS-CoV-2. *JAMA Network Open*.

[4] Yasuhara J, Watanabe K, Takagi H, Sumitomo N, Kuno T (2021). COVID-19 and multisystem inflammatory syndrome in children: A systematic review and meta-analysis. *Pediatric Pulmonology*.

[5] Son MBF, Murray N, Friedman K, Young CC, Newhams MM, Feldstein LR (2021). Multisystem Inflammatory Syndrome in Children - Initial Therapy and Outcomes. *The New England journal of medicine*.

[6] Filippatos F, Tatsi EB, Michos A (2021). Immune response to SARS-CoV-2 in children: A review of the current knowledge. *Pediatric Investigation*.

[7] Esteve-Sole A, Anton J, Pino-Ramirez RM, Sanchez-Manubens J, Fumadó V, Fortuny C (2021). Similarities and differences between the immunopathogenesis of COVID-19-related pediatric multisystem inflammatory syndrome and Kawasaki disease. *The Journal of Clinical Investigation*.

[8] Kurup S, Burgess R, Tine F, Chahroudi A, Lee DL (2022). SARS-CoV-2 Infection and Racial Disparities in Children: Protective Mechanisms and Severe Complications Related to MIS-C. *Journal of Racial and Ethnic Health Disparities*.

[9] Moreews M, Le Gouge K, Khaldi-Plassart S, Pescarmona R, Mathieu AL, Malcus C (2021). Polyclonal expansion of TCR Vbeta 21.3^+^ CD4^+^ and CD8^+^ T cells is a hallmark of Multisystem Inflammatory Syndrome in Children. *Science Immunology*.

[10] Consiglio CR, Cotugno N, Sardh F, Pou C, Amodio D, Rodriguez L (2020). The Immunology of Multisystem Inflammatory Syndrome in Children with COVID-19. *Cell*.

[11] Bohn MK, Yousef P, Steele S, Sepiashvili L, Adeli K (2022). MultiInflammatory Syndrome in Children: A View into Immune Pathogenesis from a Laboratory Perspective. *The Journal of Applied Laboratory Medicine*.

